# Electricity generation from rice bran in microbial fuel cells

**DOI:** 10.1186/s40643-016-0129-1

**Published:** 2016-11-23

**Authors:** Shu Takahashi, Morio Miyahara, Atsushi Kouzuma, Kazuya Watanabe

**Affiliations:** 1 School of Life Science, Tokyo University of Pharmacy and Life Sciences, Tokyo, 192-0392 Japan; 2 Meidensha Corporation, Shinagawa, Tokyo 141-8616 Japan

**Keywords:** Rice bran, Exoelectrogen, Pyrosequencing, *Lactobacillales*, *Desulfuromonadales*

## Abstract

**Background:**

Rice bran is a by-product of the rice milling process and mostly discarded in Japan. Although many studies have shown that microbial fuel cells (MFCs) are able to generate electricity from organic wastes, limited studies have examined MFCs for generating electricity from rice bran.

**Findings:**

Laboratory-scale single-chamber MFCs were inoculated with paddy field soil and supplied with rice bran for examining electricity generation. Power outputs and microbiome compositions were compared between MFCs containing pure water as the liquid phase (MFC-W) and those containing mineral solution (MFC-M). Polarization analyses showed that both MFCs successfully generated electricity with the maximum power densities of 360 and 520 mW m^−2^ (based on the projected area of anode) for MFC-W and MFC-M, respectively. Amplicon-sequencing analyses revealed that *Trichococcus* and *Geobacter* specifically occurred in anode biofilms in MFC-W and MFC-M, respectively.

**Conclusions:**

The results suggest that rice bran is a feasible fuel by itself for generating electricity in MFCs.

Rice bran is a by-product of the rice milling process and made of brown outer layers of rice grains. In Japan, approximately 630 million tons of rice crop are produced annually, of which rice bran makes up approximately 10% (Matsuoka and Tanaka 2013). Currently, approximately 40% of rice bran is used for oil production and 15% for cattle feeds and fertilizers, while the remaining is not specifically used and mostly discarded (Matsuoka and Tanaka 2013). In addition, since rice bran is known to be rich in vitamins and minerals (Faria et al. 2012), a small portion has been used for nutritional supplements and cosmetics. Still, a large portion of rice bran is currently discarded in Japan.

Microbial fuel cells (MFCs) are devices that use living microbes as anode catalysts for generating electricity from organic matter and have attracted social attention due to their ability to generate electricity from waste biomass and wastewater (Logan et al. 2006). For instance, MFCs have been examined for electricity generation from waste sludge (Jiang et al. 2009), cattle manure (Inoue et al. 2013), domestic wastewater (Min and Logan 2004), and brewery wastewater (Feng et al. 2008). In addition, several studies have examined the use of rice bran as a component of mixed waste substrates for electricity generation in MFCs (Moqsud et al. 2013; Schievano et al. 2016). However, no study has examined the potential of rice bran as a fuel for electricity generation in MFCs. In the present study, MFCs were operated with rice bran as the sole substrate, and power outputs and microbiome compositions were analyzed. Rice bran was suspended either in pure water or in mineral solution to check if additional nutrients are necessary for electricity generation from rice bran.

The present study used single-chamber MFCs (approximately 15 mL in capacity) that were equipped with anodes (5 cm^2^ in the projected area) and cathodes (5 cm^2^ in the projected area). An anode was made of graphite felt (GF-80-3F, Sohgoh Carbon), and a cathode was an air cathode that had four polytetrafluoroethylene layers on one side and a platinum catalyst layer (0.2 mg platinum cm^−2^; TEC10E20TPM, Tanaka Kikinzoku Kogyo) on the other side (Cheng et al. 2006). A filter paper (No. 1004-240, GE Healthcare) was sandwiched between the anode and the cathode to prevent them from making contact. An MFC was filled with 15 mL of pure water (MFC-W) or mineral solution (MFC-M), inoculated with 0.3 g of rice paddy field soil (obtained at Noda, Chiba, Japan) and supplemented with 0.1 g of rice bran as the sole organic substrate. The mineral solution contained (per liter) 0.14 g of KH_2_PO_4_, 2.5 g of NaHCO_3_, 0.54 g of NH_4_Cl, 0.20 g of MgCl_2_·6H_2_O, 0.15 g of CaCl_2_·2H_2_O, 1 mL of a trace element solution, and 2 mL of a vitamin solution (DSMZ medium 318, Deutsche Sammlung von Mikroorganismen und Zellkulturen GmbH, Germany). This solution has been used in previous studies, in which MFCs successfully generated electric power (Miyahara et al. 2016a, b). We also examined MFC without adding the mineral nutrients described above (MFC-W), since it has been suggested that rice bran is rich in vitamins and minerals (Faria et al. 2012). We though that electricity generation without nutritional additives would be desirable for reducing costs for the treatment of waste rice bran.

After MFC was filled with the electrolyte (pure water or the mineral solution) containing paddy soil and rice bran, it was put in an incubator at 30 °C. The operation was initiated by connecting the anode and the cathode via an external resistor (10,000 Ω), and a voltage (*E*) across the resistor was monitored using a data logger (GL800, Graphtec). When the voltage dropped down to below 0.1 V, rice bran (0.1 g) was added to recover electric output. MFC performances were evaluated by polarization analyses using a potentiostat (HSV-100, Hokuto Denko) as described previously (Watanabe 2008). The maximum power density (the peak in a power curve, *P*_max_) was determined based on the projected area of anode (mW m^−2^). Protein contents in anode biofilms, cathode biofilms, and electrolyte suspensions were determined to assess the amounts of microbes present there as described previously (Shimoyama et al. 2009). Phylogenetic compositions of bacteria in anode biofilms, cathode biofilm, and electrolyte suspension were analyzed by pyrosequencing of PCR-amplified 16S rRNA gene fragments. DNA extraction, PCR amplification, and amplicon purification were conducted as described elsewhere (Miyahara et al. 2013). Amplicons from different samples were mixed at a same concentration (1 ng μl^−1^ each) and subjected to pyrosequencing using a Genome Sequencer FLX system (Miyahara et al. 2013). Phylogenetic analyses were conducted using a DDBJ 16S rRNA database, a blastn program (Altschul et al. 1997), and an RDP classifier (Wang et al. 2007). Nucleotide sequences determined in the present study were deposited into the DDBJ Sequence Read Archive Database (accession number: DRA005155).

MFC-W and MFC-M were operated for approximately 130 days (Fig. 1), and, during the operation, rice bran was added when *E* dropped down to below 0.1 V (once every 10–20 days). After an initial acclimatization period (approximately 30 days), the addition of rice bran resulted in immediate increases of *E* up to over 0.5 V. We conducted the polarization analysis when *E* was over 0.5 V, and typical polarization and power curves are shown in Fig. 2a. The average values of *P*_max_ were compared between MFC-W and MFC-M (Fig. 2b), showing that MFC-M generated significantly higher outputs than MFC-W. However, it is also noteworthy that MFC-W (containing only rice bran) generated the substantial level of output, suggesting that rice bran is a feasible substrate by itself for generating electricity in MFCs. In MFCs, microbes oxidize organic matter and release electrons that are transferred to anodes, resulting in electricity generation (Watanabe 2008). The present study shows that rice bran is a potent organic substrate. In addition, it is likely that minerals and vitamins contained in rice bran stimulated the growth of microbes involved in electricity generation in MFCs.

**Fig. 1:**
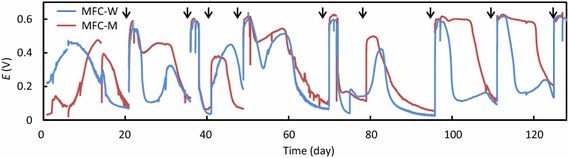
Changes in *E* values during the operation of MFC-W and MFC-M. Rice bran was added at time points indicated with *arrows*

**Fig. 2:**
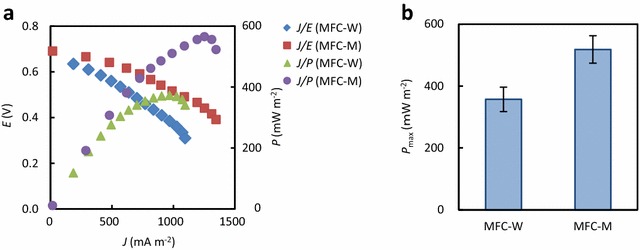
Evaluation of MFCs fueled by rice bran. **a** Representative polarization (*J/E*) and power (*J/P*) curves for MFC-W and MFC-M. **b** A comparison of *P*_max_ between MFC-W and MFC-M. *Datum bars* indicate mean values and *error bars* indicate SD (*n* = 3)

We were interested in analyzing microbes involved in electricity generation from rice bran. As the initial step, the amounts of microbes in anode biofilms, cathode biofilms, and electrolyte suspensions in MFC-W and MFC-M as assessed by the protein contents were compared (Fig. 3). We found that, in addition to the anodes, substantial amounts of microbes also attached to the cathodes. Microbes of the cathodes are considered to respire oxygen that entered from the atmosphere through the air–cathode membranes. In addition, microbes were also present in the electrolyte suspensions, and these may have included fermentative microbes that decomposed rice bran. Comparisons in microbiomes in these habitats were therefore expected to facilitate the identification of microbes responsible for electricity generation at the anodes.

**Fig. 3:**
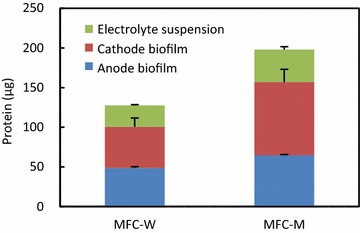
Protein contents in MFC-W and MFC-M. The total amount of protein in a MFC is the sum of protein contents in anode biofilm, cathode biofilm, and planktonic cells in electrolyte

Phylogenetic compositions of bacteria in anode biofilms, cathode biofilms, and electrolyte suspensions in MFC-W and MFC-M were determined by the sequence analyses of amplified 16S rRNA gene fragments (Fig. 4). We focused on bacteria, since all known exoelectrogens (electricity-generating microbes) are affiliated with the *Bacteria* (Kumar et al. 2015). Figure 4a shows that the orders *Bacteroidales* and *Clostridiales* were ubiquitously found in all samples, while some taxa were specifically detected. For instance, the *Desulfuromonadales* specifically occurred in the anode biofilm in MFC-M, while the *Burkholderiales* increased in the cathode biofilms in MFC-W and MFC-M. In addition, the *Lactobacillales* substantially increased in the anode biofilm in MFC-W.

**Fig. 4:**
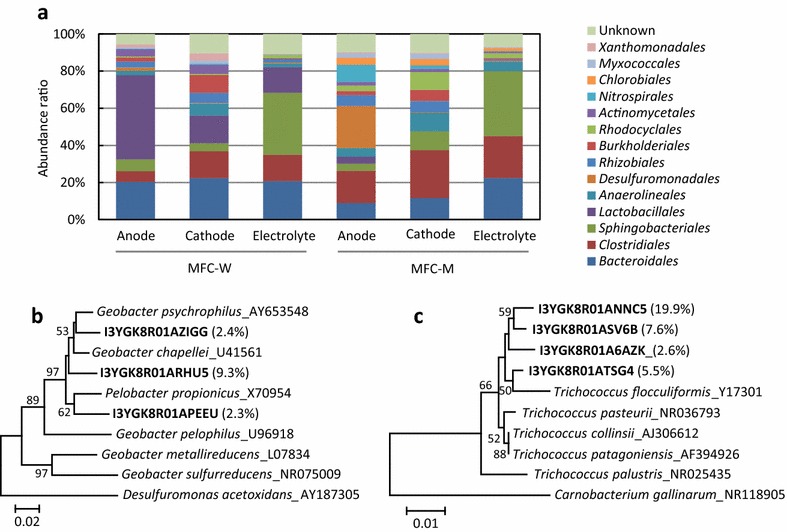
Phylogenetic analyses of bacteria in anode biofilm, cathode biofilm, and electrolyte in MFC-W and MFC-M. **a** Phylogenetic distribution of bacteria in each sample. **b** Phylogenetic positions of major sequences in the anode biofilm in MFC-M affiliated with the genus *Geobacter*. **c** Phylogenetic positions of major sequences in the anode biofilm in MFC-W affiliated with the genus *Trichococcus*. In **b** and **c**, percentages to the total sequence are shown in parentheses, and numbers at branch nodes are bootstrap values per 100 trials

Based on the characteristic distributions and currently available physiological information on members of these orders (Krieg 2011; Wiegel et al. 2006), it is considered that the *Bacteroidales* and *Clostridiales* bacteria fermentatively decomposed rice bran in these MFCs. On the other hand, since the family *Desulfuromonadales* is known to include exoelectrogens, such as those affiliated with the genera *Geobacter* (Lovley et al. 2011) and *Desulfuromonas* (Alves et al. 2011), it is reasonable to consider that the *Desulfuromonadales* bacteria specifically detected in the anode biofilm in MFC-M were responsible for electricity generation. In order to more precisely identify phylogenetic positions of the *Desulfuromonadales* bacteria detected in MFC-M, major *Desulfuromonadales* sequences (over 2% to the total) were used to construct a phylogenetic tree (Fig. 4b). This analysis confirmed that the major members of the *Desulfuromonadales* are affiliated with the genus *Geobacter*.

Major *Lactobacillales* sequences detected in the anode biofilm in the MFC-W were also analyzed (Fig. 4c), and we found that they are affiliated with the genus *Trichococcus* (Liu et al. 2002). This genus has been described for aerotolerant fermentative organisms that had been isolated from aerobic sludge and anaerobic sediment (Liu et al. 2002). Although no previous studies have identified exoelectrogens that are affiliated with this genus, *Trichococcus* has been detected in association with the biostimulation of anaerobic digestion with (semi)conductive ferric oxides involving direct interspecies electron transfer (Baek et al. 2015). Since *Trichococcus* was highly enriched in the anode biofilm in MFC-W, we suggest that *Trichococcus* bacteria detected in the anode biofilm are capable of utilizing electrodes as extracellular electron acceptors. It should also be noted that anode microbiomes (including exoelectrogens) changed whether or not the electrolyte was supplemented with the inorganic nutrient components. We deduce that *Trichococcus*, a genus of lactic acid bacteria, thrives in association with rice bran and adapts to a situation where rice bran is the sole nutrient for growth.

In conclusion, we suggest that rice bran is a feasible fuel for electricity generation in MFCs. Since substantial outputs were generated in MFC-W (rice bran was suspended in pure water), we suggest that rice bran contains sufficient nutrients for electricity generation by microbes. However, it is also shown that the supplementation with the inorganic nutrient solution improves electric output from rice bran. In future studies, the utility of rice bran will be further evaluated in large-scale MFC reactors with different configurations and electrodes.

## References

[CR1] Altschul SF, Madden TL, Schaffer AA, Zhang J, Zhang Z, Miller W, Lipman DJ (1997). Gapped BLAST and PSI-BLAST: a new generation of protein database search programs. Nucleic Acids Res.

[CR2] Alves AS, Paquete CM, Fonseca BM, Louro RO (2011). Exploration of the ‘cytochrome’ of *Desulfuromonas acetoxidans*, a marine bacterium capable of powering microbial fuel cells. Metallomics.

[CR3] Baek G, Kim J, Cho K, Bae H, Lee C (2015). The biostimulation of anaerobic digestion with (semi) conductive ferric oxides: their potential for enhanced biomethanation. Appl Microbiol Biotechnol.

[CR4] Cheng S, Liu H, Logan BE (2006). Increased performance of single-chamber microbial fuel cells using an improved cathode structure. Electrochem Commun.

[CR5] Faria SASC, Bassinello PZ, Penteado MVC (2012). Nutritional composition of rice bran submitted to different stabilization procedures. Braz J Pharm Sci.

[CR6] Feng Y, Wang X, Logan BE, Lee H (2008). Brewery wastewater treatment using air-cathode microbial fuel cells. Appl Microbiol Biotechnol.

[CR7] Inoue K, Ito T, Kawano Y, Iguchi A, Miyahara M, Suzuki Y, Watanabe K (2013). Electricity generation from cattle manure slurry by cassette-electrode microbial fuel cells. J Biosci Bioeng.

[CR8] Jiang J, Zhao Q, Zhang J, Zhang G, Lee DJ (2009). Electricity generation from biotreatment of sewage sludge with microbial fuel cell. Bioresour Technol.

[CR9] Krieg NR, Krieg NR, Staley JT, Brown DR, Hedlund BP, Paster BJ, Ward NL, Ludwig W, Whitman WB (2011). Order I. *Bacteroidales* ord. nov. Bergey’s Manual of Systematic Bacteriology.

[CR10] Kumar R, Singh L, Wahid ZA, Din MFM (2015). Exoelectrogens in microbial fuel cells toward bioelectricity generation: a review. Int J Energy Res.

[CR11] Liu JR, Tanner RS, Schumann P, Weiss N, McKenzie CA, Janssen PH, Seviour EM, Lawson PA, Allen TD, Seviour RJ (2002). Emended description of the genus *Trichococcus*, description of *Trichococcus collinsii* sp. nov., and reclassification of *Lactosphaera pasteurii* as *Trichococcus pasteurii* comb. nov. and of *Ruminococcus palustris* as *Trichococcus palustris* comb. nov. in the low-G + C gram-positive bacteria. Int J Syst Evol Microbiol.

[CR12] Logan BE, Hamelers B, Rozendal R, Schröder U, Keller J, Freguia S, Aelterman P, Verstraete W, Rabaey K (2006). Microbial fuel cells: methodology and technology. Environ Sci Technol.

[CR13] Lovley DR, Ueki T, Zhang T, Malvankar NS, Shrestha PM, Flanagan KA, Aklujkar M, Butler JE, Giloteaux L, Rotaru AE, Holmes DE, Franks AE, Orellana R, Risso C, Nevin KP (2011). *Geobacter*: the microbe electric’s physiology, ecology, and practical applications. Adv Microb Physiol.

[CR14] Matsuoka H, Tanaka S (2013). Enhancement antioxidant effect of the fermented rice bran and isolation of the antioxidative component. Mukai-zaidan Rep..

[CR15] Min B, Logan BE (2004). Continuous electricity generation from domestic wastewater and organic substrates in a flat plate microbial fuel cell. Environ Sci Technol.

[CR16] Miyahara M, Hashimoto K, Watanabe K (2013). Use of cassette-electrode microbial fuel cell for wastewater treatment. J Biosci Bioeng.

[CR17] Miyahara M, Kouzuma A, Watanabe K (2016). Sodium chloride concentration determines exoelectrogens in anode biofilms occurring from mangrove-grown brackish sediment. Bioresour Technol.

[CR18] Miyahara M, Sakamoto A, Kouzuma A, Watanabe K (2016). Poly iron sulfate flocculant as an effective additive for improving the performance of microbial fuel cells. Bioresour Technol.

[CR19] Moqsud MA, Omine K, Yasufuku N, Hyodo M, Nakata Y (2013). Microbial fuel cell (MFC) for bioelectricity generation from organic wastes. Waste Manag.

[CR20] Schievano A, Sciarria TP, Gao YC, Scaglia B, Salati S, Zanardo M, Quiaob W, Dongb R, Adani F (2016). Dark fermentation, anaerobic digestion and microbial fuel cells: an integrated system to valorize swine manure and rice bran. Waste Manag.

[CR21] Shimoyama T, Yamazawa A, Ueno Y, Watanabe K (2009). Phylogenetic analyses of bacterial communities developed in a cassette-electrode microbial fuel cell. Microbes Environ.

[CR22] Wang Q, Garrity GM, Tiedje JM, Cole JR (2007). Naive bayesian classifier for rapid assignment of rRNA sequences into the new bacterial taxonomy. Appl Environ Microbiol.

[CR23] Watanabe K (2008). Recent developments in microbial fuel cell technologies for sustainable bioenergy. J Biosci Bioeng.

[CR24] Wiegel J, Tanner R, Rainey FA, Dworkin MM, Falkow S, Rosenberg E, Schleifer KH, Stackebrandt E (2006). An introduction to the family Clostridiaceae. The prokaryotes.

